# Proteins with two SUMO-like domains in chromatin-associated complexes: The RENi (Rad60-Esc2-NIP45) family

**DOI:** 10.1186/1471-2105-6-22

**Published:** 2005-02-07

**Authors:** Maria Novatchkova, Andreas Bachmair, Birgit Eisenhaber, Frank Eisenhaber

**Affiliations:** 1 Gregor Mendel-Institut GMI, Austrian Academy of Sciences, Vienna Biocenter, A-1030 Vienna, Austria; 2 Research Institute of Molecular Pathology, Dr. Bohr-Gasse 7, A-1030 Vienna, Austria; 3 Max Planck Institute for Plant Breeding Research, Carl-von-Linné-Weg 10, D-50829 Cologne, Germany

## Abstract

**Background:**

Post-translational modification by Small Ubiquitin-like Modifiers (SUMO) has been implicated in protein targeting, in the maintenance of genomic integrity and in transcriptional control. But the specific molecular effects of SUMO modification on many target proteins remain to be elucidated. Recent findings point at the importance of SUMO-mediated histone NAD-dependent deacetylase (HDAC) recruitment in transcriptional regulation.

**Results:**

We describe the RENi family of SUMO-like domain proteins (SDP) with the unique feature of typically containing two carboxy-terminal SUMO-like domains. Using sequence analytic evidence, we collect family members from animals, fungi and plants, most prominent being yeast **R**ad60, **E**sc2 and mouse **NI**P45 . Different proteins of the novel family are known to interact directly with histone NAD-dependent deacetylases (HDACs), structural maintenance of chromosomes (SMC) proteins, and transcription factors. In particular, the highly non-trivial designation of the first of the two successive SUMO-domains in non-plant RENi provides a rationale for previously published functionally impaired mutant variants.

**Conclusions:**

Till now, SUMO-like proteins have been studied exclusively in the context of their covalent conjugation to target proteins. Here, we present the exciting possibility that SUMO domain proteins, similarly to ubiquitin modifiers, have also evolved in a second line – namely as multi-domain proteins that are non-covalently attached to their target proteins. We suggest that the SUMO stable fusion proteins of the RENi family, which we introduce in this work, might mimic SUMO and share its interaction motifs (in analogy to the way that ubiquitin-like domains mimic ubiquitin). This presumption is supported by parallels in the spectrum of modified or bound proteins e.g. transcription factors and chromatin-associated proteins and in the recruitment of HDAC-activity.

## Background

Among ubiquitin-related proteins, containing at least one domain with a ubiquitin-like fold, one can distinguish ubiquitin-like modifiers (UBLs) and ubiquitin domain proteins (UDPs) [1].

UBLs can be covalently attached to target proteins analogously to ubiquitin. Unlike ubiquitin, UBLs mostly do not directly target proteins for degradation [2], although functional links can exist. One of the most heavily researched single domain UBLs, the small ubiquitin-related modifier (SUMO), is known to act on transcription factors, chromatin associated proteins, nuclear body proteins and septins [2].

In contrast to UBLs, UDPs are not conjugated to other proteins and lack the C-terminal double glycine motif characteristic for ubiquitin and ubiquitin-like modifiers. They are a heterogeneous class of usually multi-domain proteins, which are unrelated outside of their ubiquitin-like domain [1]. In several cases, it has been demonstrated that the ubiquitin-domain within those proteins likely fulfills its cellular role by functionally mimicking ubiquitination [3,4].

The biological relevance of non-conjugatable multi-domain proteins having a domain with clear relationship to UBLs like SUMO, rather than ubiquitin, is yet unknown. Here, we present a detailed sequence analysis of a family of SUMO-like domain proteins (SDPs) containing one or two SUMO-like domains. Members of the proposed RENi family act as factors in transcriptional regulation, chromatin silencing and genomic stability.

## Results

### Sequence architecture of *Drosophila melanogaster *CG4449

During the study of the predicted nuclear subset of the Drosophila proteome, we encountered the unknown 424 amino acids long protein CG4449 (NP_651134). Initial analysis of its sequence complexity shows that the disordered N-terminal half of the protein is followed by a likely globular segment (predicted using Pdisorder by Softberry, Inc). Indeed, a compositionally biased, polar low-complexity region (LCR) spans almost the entire N-terminal 220 amino acids (AA) as reported by CAST (region 47–165, lysine-rich) [5] and SEG (regions 46–77 and 133–178, parameters 25/3.0/3.3) [6].

The C-terminal half of CG4449 turns out to contain an internally repeated segment identifiable with RADAR [7] (region 270–309 matching 368–407). In an attempt to confirm this repeat, we queried the protein against the conserved domain database using RPS-BLAST [8]. Thereby, we could define a similarity to SUMO-like domains overlapping with the second repeat-element (see Table 1 for details), while no significant hits emerged for the first of the repeat-constituents. Using profile-profile comparison, however, segment 220–325 is shown to possess a distant, yet significant similarity to SUMO sequences and, therefore, to share the SUMO fold (Table 1).

In conclusion, we found that the Drosophila protein CG4449 (NP_651134) has a tripartite architecture: with a N-terminal LCR followed by two globular domains with a SUMO-like fold (termed SD1 and SD2). Whereas SD2's similarity to single domain SUMO-like sequences can be easily detected with BLAST tools, the identification of SD1 is non-trivial (Table 1). For both SD1 and SD2, a carboxy-terminal double-glycine motif, as it is known and necessary for the covalent attachment of SUMO proteins, is missing. This finding is remarkable as SUMO proteins are discussed in the scientific community solely as polypeptides that become covalently bonded to various targets [9]. Here, we present cases of non-conjugatable poly-SUMO fusion protein.

### Collecting animal NIP45-related proteins characterized by two SUMO-like domains

A PSI-BLAST search started with the globular C-terminal half of CG4449 (220–424) including the two SUMO-like domains, collects a family of animal proteins with the same tripartite organization in *C. briggsae *(CAE71155.1, E = 0.001 round 2), *H. sapiens *(NP_116204.2, E = 0.003 round 2), *M. musculus *(NP_035030, E = 1e-39 round 3) and *C. elegans *(NP_497960, E = 2e-12 round 3). All these proteins contain a LCR at the N-terminus followed by two SUMO-like domains, the first of which has mostly diverged away beyond recognition thresholds using traditional sequence-profile searches (Table 1). The human and the mouse homologs correspond to the studied nuclear factor NIP45 (NF-AT interacting protein) [10]. All sequences and original database search results can be found at the RENi homepage [11].

### Distant NIP45 homologs in fungi, other lower eukaryotes and plants

#### Indications on the existence of NIP45 homologs in lower eukaryotes and plants

**Figure 1:**
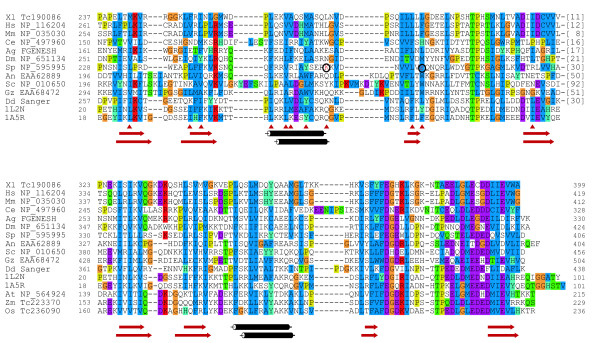
**Multiple Sequence Alignment of Rad60-Esc2-NIP45 (RENi) members. **The alignment is CLUSTAL colored [44]. The organism from which a sequence has been derived is indicated by a 2 letter code preceding the database accession numbers: An *Aspergillus nidulans*, Ag *Anopheles gambiae*, At *Arabidopsis thaliana*, Ce *Caenorhabditis elegans*, Dd *Dictyostelium discoideum*, Dm *Drosophila melanogaster*, Gz *Gibberella zeae*, Hs *Homo sapiens*, Mm *Mus musculus*, Os *Oryza sativa*, Sc *Saccharomyces cerevisiae*, Sp *Schizosaccharomyces pombe*, Xl *Xenopus laevis*, Zm *Zea mays*. All accession numbers can be found in the NCBI non-redundant database, except 1) the ones of Xl, Zm and Os, which correspond to the TIGR Contig identifiers from which a presumptive translation was derived 2) the Dd protein which is included in the Sanger protein set with the accession JC3V1_0C0008_11033 3) the Ag protein was derived using FGENESH on a segment from Anopheles gambiae str. PEST chromosome 2L (accession AAAB01008810) 4) IL2N, 1A5R are entries from the PDB. The two alignment blocks correspond to the two distinct SUMO-like domains in RENi proteins. Only the second block includes plant representatives, which seem to miss the first SUMO-like domain. The SUMO sequences IL2N, 1A5R have been aligned to both blocks and the secondary structure elements below the alignment are derived from the PDBsum entries for both of these. Triangles mark positions reported to be involved in maintaining the ubiquitin-fold of human SUMO-1 [21]. These are also highlighted in the structural representation in Figure 2. Black encycled red-colored residues in NP_595995 point at the mutations in the rad60-1 (K263E) [15] and rad60-3 (F272V) [20] variants.

A multiple sequence alignment of the globular C-terminal half of *D. melanogaster *CG4449 (220–424) and the corresponding sequences derived from *A. gambie*, *X. laevis*, *C. elegans*, *C. briggsae*, *M. musculus *and *H. sapiens *(Figure 1) was used to generate a Hidden Markov Model (HMM; in the global alignment mode). The protein family was enlarged using the HMMER2 tool [12] in searches against single model organism proteomes. These searches retrieved as best hits in the respective proteomes the likely homologs in *D. discoideum *(Sanger proteome identifier- JC3V1_0C0008_11033, 0.00059) *A. thaliana *(At1g68185.1, NP_564924.1, E = 0.00076), *O. sativa *(NP_917594.1, E = 0.0019), *S. pombe *(NP_595995.1, E = 0.00073), *S. cerevisiae *(NP_010650.1, E = 0.11), *Y. lipolytica *(CAG82446, E = 0.00087), *C. glabrata *(CAG57776, E = 0.52) and in other recently published fungi proteomes [13]. The *S. cerevisiae *and *S. pombe *homologs correspond to the studied Rad60 and Esc2 proteins, respectively [14,15]; the remaining proteins are uncharacterized.

These HMM-search results suggest the most likely plant and lower eukaryote orthologues to the animal NIP45-like proteins. For establishing the orthology relationship, these initial results need to be confirmed by reciprocal searches independently performed for fungal and plant proteins. Further below, we present this evidence for the homology between the C-terminal part of the proteins found in the various taxonomic groups.

#### Confirming fungal family members in reciprocal searches

The set of fungal RENi proteins can be autonomously collected using a BLASTP search started with the *C. glabrata *representative (CAG57776) and retrieving the best and significant hits in the proteomes of *S. cerevisiae*, *S. pombe*, *K. lactis*, *C. albicans*, *Y. lipolytica*, *D. hansenii*, *A. nidulans *[11]. The domain architecture of these fungal homologs is likely also tripartite (Figure 1, Table 1). It differs from the animal representatives by a longer sequence separating the two SUMO-like domains (many dozens of residues compared with ~10 in the case of animal proteins), which is typically of highly helical content (determined using NPL consensus secondary structure prediction [16]). A HMM was generated from a multiple sequence alignment of the SUMO-domain containing C-terminal half of the listed fungal homologs, where gap only columns replaced the compositional biased helical region between the two SUMO domains. A search with this HMM retrieved as best hits in the respective proteomes the RENi proteins in *M. musculus *(NP_035030, E = 0.005), *A. thaliana *(NP_564924, E = 0.016), *C. elegans *(NP_497960, E = 0.0012).

#### Confirming plant family members in reciprocal searches

Potential plant RENi homologs, derived in a full-length TBLASTN search with *A. thaliana *(At1g68185.1, NP_564924.1) against the TIGR Gene indices of barley, maize, rice, potato and soybean [17], show a length of 210–240 AA and are thus around 100 AA shorter than the shortest animal homolog from worm. The domain organization seems also to be distinct. A 100 AA N-terminal, very polar region (with two conserved motifs E [ED]LEPLFDY [SR]RVQP and DWLPPPP found with MEME [18]) is followed by ~40 AA with predicted strong helical preference (using NPL [16]) and a clear C-terminal SUMO-like domain (Table 1). There are no indications for another SUMO-like domain at the N-terminal side of the ~40 AA helical region. Further confirmation of the relationship between the listed RENi proteins of the Viridiplantae and Fungi/Metazoan group comes from the analysis of the reciprocal genomic best hits of *A. thaliana *in *Y. lipolytica *(At vs Yl 1e-07, Yl vs At 6e-04) and *H. sapiens *proteomes (At vs Hs 3e-05, Hs vs At 8e-05) [11].

### Definition of the Rad60-Esc2-NIP45 (RENi) protein family

We propose to name the collected group of protein sequences the RENi-family after its most studied members Rad60, Esc2 and NIP45. All representatives have a similar sequence architecture involving a N-terminal low complexity region with many polar and (positively) charged residues and a C-terminal globular part with one (plant proteins) or two (all others) SUMO-like domains.

The use of a model representing the complete globular region of RENi proteins was essential for the successful collection of the family. A global HMM spanning the SD1 and SD2 domains tests for homology in the whole globular part and, correspondingly, directly collects the RENi family. In contrast, when using the C-terminal half of various RENi family members as query sequence in PSI-BLAST [19], the searches are invaded by SUMO proteins (hitting only the segment of SD2) before the RENi family can be collected. This means that the SD2 domain sequence segments of the RENi group and the family of single-domain SUMO proteins are not well separated in sequence space (Figure 3). For this reason, the similarity of NIP45, Esc2 and Rad60 could previously only been defined transitively via the similarity of their SD2 segment to SUMO proteins and their similar length [20].

## Discussion

### The SUMO-like domains in proteins of the Rad60-Esc2-NIP45 (RENi) family

While RENi proteins of the fungal, metazoan and mycetozoan taxa contain two C-terminal SUMO-like domains (SD1 and SD2), only the second one can be clearly defined in plant representatives (Table 1, Figure 4). This very C-terminal SD2 domain, shares several features discriminating SUMO proteins from other ubiquitin-like modifiers, as for example the large negative charged cluster, seen in the alignment 5–15 residues from the very C-terminus (Figure 1). The negative surface patch formed by these residues has been suggested to shape a SUMO-typical interaction surface [21]. RENi proteins lack conservation of the carboxy-terminal double-glycine motif required for covalent attachment of SUMO to its substrates. Thus, they are likely linear non-cleavable SUMO fusions, which cannot be conjugated to target proteins, and have to be classified as UDPs.

The SD1 SUMO-like domain contained in fungi, metazoa and mycetozoa, has sequentially diverged away from SUMO proteins, but structural prediction suggest its resemblance to SUMO (Table 1). The low sequence conservation of this domain does not understate a possible functional conservation in that region, as it has been shown that the structure rather than sequence is important for the function of ubiquitin-like domains (UD). For example, replacing the UD of the UDP Rad23 with ubiquitin renders a functional protein variant [1].

**Figure 2:**
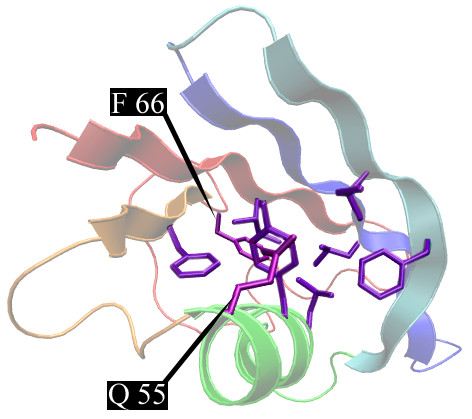
**Ribbon diagram of the human SUMO-1 protein structure **[21]. The displayed region corresponds to the segment shown in the alignment in Figure 1. The core residues L24, I34, F36, F64, F66, I88 located in the beta-strands and L44, L47, K48, Y51, Q55 in the helix form critical contacts in the helix-sheet interface of the SUMO core. These residues are shown in the stick mode (in purple). The highlighted Q55 and F66 residues align with residues mutated in the rad60-1 (K263E) [15] and rad60-3 (F272V) [20] variants (see also Figure 1).

Indications on the functional importance of the first SUMO-like domain in RENi proteins come from the two fungal representatives of the family. The fission yeast rad60-1 (K263E) [15] and rad60-3 (F272V) [20] mutants, which are defective in the rad60 function of double strand break repair, contain a point-mutation within this first SUMO-like domain (SD1). The sequence alignment to human SUMO-1 (structure 1A5R, see Figure 1) indicates that both mutations most likely affect structurally important positions. They align to residues within human SUMO-1 (1A5R) (Gln55 and Phe66) that have been listed by Bayer et al. [21] among the contacts contributing with parts of their side chain to the formation of the hydrophobic core of the fold (Figure 1 and Figure 2). In the budding yeast Esc2p, the region containing the first SUMO-domain SD1 together with a 80 AA low-complex N-terminal segment can be defined as a sufficient fragment supplying its function in targeted silencing (residues 115–389 in Esc2p) [14].

**Figure 3:**
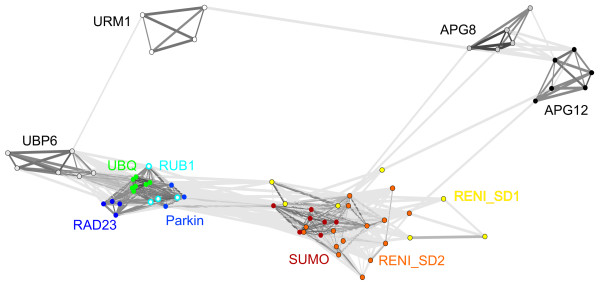
**Graph representation of pair-wise sequence similarities for known ubiquitin-like domains plus SD1 and SD2 domains. **SD1 and SD2 domains cluster with SUMO proteins. The graph was generated using CLANS [22] and shows all pairwise interactions based on HSP P-values calculated using all-against-all BLASTP. The sequence file used as an input is available on the homepage [11]. Analysis of a more extensive assembly of ubiquitin-fold proteins [45] leads to the same conclusion, but a less well readable graph (not shown). AGP8 – autophagy 8 like proteins, AGP12 – autophagy 12 like proteins, URM1 – ubiquitin related modifier 1, UBP6 - Ubiquitin-specific processing protease 6, RUB1 – Related to ubiquitin 1.

**Figure 4:**
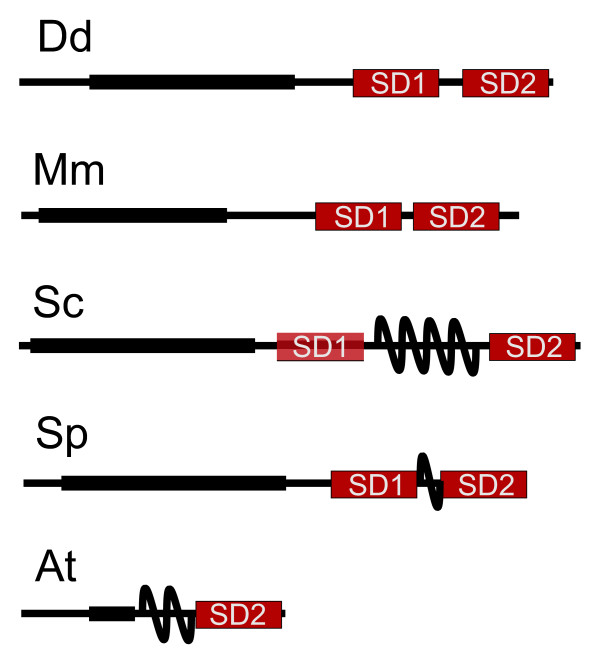
**Domain architecture of RENi proteins. **Red boxes: SUMO-related domains (labeled SD1 and SD2 respectively), black thick lines: low complexity regions. Species code and accession numbers as in Figure 1. Black waves indicate long helical regions (>20 AA), conserved among close relatives.

A graph representation of the pair-wise similarity relationship for SD1 and SD2 sequences to other known ubiquitin-like domains (Figure 3, created with the program Clans [22]) illustrates that both are most closely related to SUMO domains. From our analysis of sequence similarity, we suggest that, at least, the very C-terminal SUMO-like domain (SD2) in RENi proteins is able to mimic SUMO and potentially shares its interaction partners. On the other hand, the available experimental data confirms the functional importance of the SUMO-like domain SD1 preceding it.

### The N-terminal polar low complexity region in proteins of the Rad60-Esc2-NIP45 (RENi) family

The occurrence of a N-terminal low complexity region with an excess of polar/charged residues is a characteristic element of the RENi protein architecture. Most likely, this is a conformationally flexible segment without inherent structural preference [23,24]. The molecular function of this region remains unknown. It should also be noted that homology considerations are not applicable to such compositionally biased regions for functional prediction.

Boddy et al. [20] discussed the possible existence of coiled coils in the domain architecture of Rad60, Esc2 and NIP45. We found that the COILS [25] tool generates hits only in few representatives of the RENi family. They are positionally not conserved relative to the two SUMO-like domains. It is known that the COILS tool produces a considerable number of false-positive hits, especially in regions with many polar/charged residues, for example, as is likely the case for a glutamic-acid-rich part in Rad60.

### Experimentally verified functions of RENi proteins

Functional information about RENi family members is restricted to the fungal Esc2, Rad60 and the metazoan NIP45 proteins. Here, unfortunately most of the existing data relates to the full-length sequences. Nevertheless, the quite divergent set of functions known for RENi proteins shows considerable overlap with the established cellular roles of SUMO proteins in genome replication and regulation of gene expression.

The fission yeast Rad60 protein was shown to be essential in DNA double-strand break repair, and to be critical also for normal growth [15]. It physically and genetically interacts with the Smc5/6 complex, a complex with a housekeeping role in the genome [20]. Interestingly, the Smc5/6 complex [26] also includes Nse2, a protein containing a zf-MIZ domain commonly found in E3-like SUMO ligases (Pfam-search E = 0.0074). In addition, Rad60 is known to bind the replication checkpoint kinase Cds1 [20].

*S. cerevisiae *Esc2 (establishment of silent chromatin 2) is involved in chromatin silencing via the recruitment or stabilization of the Sir (silent information regulators) complex [27,28]. It is known to interact with Sir2, a histone NAD-dependent deacetylase (HDAC-class III) of the Sir complex, which is well conserved from bacteria to human [29] and, thus, might be an interaction partner of other RENi proteins as well. Similarly to other HDACs, Sir2 proteins are recruited to chromatin by DNA-bound factors [30] and act by deacetylating histones [31] as well as transcription factors such as p53 and forkhead transcription factor (shown for hSIR2) [29,32]. With respect to a possible intersection with known Rad60 functions, it is interesting that Sir2 is not only involved in heterochromatic gene silencing and euchromatic repression [30] but also in DNA double-strand break repair mediated by end-joining [33].

NIP45, the one studied RENi in metazoa, has been implied in gene regulation, where it needs its DNA-binding partner NFATp for this activity [34]. Strikingly, the NFAT family member NFAT1 that interacts with NIP45 [10] was independently shown to be sumoylated [35]. NFAT1 sumoylation acts in nuclear retention, regulation of transcriptional activity and recruitment to nuclear SUMO-1 bodies [35]. This analysis might suggest a potential role for SUMO-like NIP45 in its complex with NFAT proteins.

### Possible functional role of the SUMO-like domains in RENi proteins

There is little experimental data on the importance of the predicted SUMO-like domains in RENi proteins. Nevertheless, all listed functions of RENi proteins conform with the known role of SUMO in transcriptional regulation and the control of genome integrity [36]. In the context of transcriptional repression, SUMO-modification has been suggested to recruit class I and II HDACs to promoter sites. Regarding genome stability, SUMOylation in DNA-repair proteins is thought to target these to DNA damage foci. The following parallels in RENi proteins become obvious: 1) HDAC recruitment has also been suggested for the fission yeast Esc2p [27,28]. 2) Mammalian NIP45 binds to transcription factors that can also be modified by SUMO [35]. 3) RENi and SUMO share a functional context in double-strand break repair and transcriptional regulation. On the basis of functional overlaps of SUMO and RENi proteins, we can speculate that RENi proteins act as SUMO stable fusion proteins "mimicking" SUMO and that they might have common interaction partners.

## Conclusions

In this report, we use sequence-analytical methods to infer the homology relationships between RENi family members and determine their tripartite (bipartite for plant homologs) domain architecture. A N-terminal polar low-complexity segment and two consecutive SUMO-like domains in the C-terminal half characterize the functionally described fungal and metazoan RENi proteins. While the more C-terminal SD2 is easily detectable, it is the particularly divergent SD1 that was shown in fungi to be essential for the assayed molecular functions. Due to the likely limited sequential- (as opposed to structural-) requirements, this SUMO-like domain is difficult to detect and has been missed in previous analyses of individual family members. The identification of the more N-terminal SUMO-like domain SD1 helps rationalizing experimental findings for mutant fungal RENi family members.

## Methods

RPS-BLAST [8] and FFAS [37] algorithms were used to search the COG [38], SCOP [39] and SMART databases [40]. SEG [6] and CAST [5] were applied in identifying low-complexity regions. Structural similarity was determined using the fold prediction methods FFAS [37] and BIOINBGU [41]. T-coffee was used for initial multiple sequence alignment [42]. CLANS [22] generated the pairwise similarity graph. VMD [43] was used for molecular visualization and POV-ray for the follow-up image rendering.

## Authors' contributions

The sequence analytic work was executed by MN. All authors (MN, AB, BE, FE) contributed to evaluating the results and making the discoveries reported here. MN prepared all the figures and, together with FE, the manuscript text. All authors read and approved the final manuscript.

## Figures and Tables

**Table 1:** Representative search hits supporting the SUMO relationship of globular segments in RENi family members. RENi family members (column 1) have been initially split into likely globular segments (column 2). These sequences have been submitted to sequence-based domain and fold-recognition methods (RPS-BLAST [8], FFAS03 [37], column 3) as well as a structure-based similarity method (INBGU [41]) (column 4). For FFAS03 scores below -9.5 are considered significant, for the Fischer fold prediction method (INBGU) it is a score of 12 and above. The following database identifiers correspond to the tabulated domains: COG5227 for the SMT3 domain in COG, smart00213 for UBQ (ubiquitin and ubiquitin-like) in SMART. The mentioned PDB identifiers represent structures of the following molecules: 1A5R Sumo-1 in human, 1L2NA Smt3, the SUMO homolog in budding yeast, 1EUVB Smt3 in budding yeast. The sequence-only similarity methods can be ordered with increasing sensitivity as: (i) RPS-BLAST, (ii) FFAS03 against a sequence database (COG), and (iii) FFAS03 against a structure database (SCOP). Only the significant hit with the lowest sensitivity method is reported for each sequence.

**Species/Accession**	**Segment**	**Method/DB/Hit/E-value**	**Hit/Score**
*D. melanogaster/ *NP_651134	220–325	FFAS03/COG/SMT3/-10.400	1A5R/27.1 1L2NA/13.6
	325–424	RPS-BLAST/COG/SMT3/9e-04	1L2NA/42.4 1A5R/24.4
*S. pombe/ *NP_595995	200–315	FFAS03/scop165/1euvb/-11.4	1L2NA/26.4 1A5R/19.7
	315–406	FFAS03/COG/SMT3/-16.3	1A5R/16.1 1L2NA/15.2
*S. cerevisiae/ *NP_010650	330–456	FFAS03/COG/SMT3/-36.6	1A5R/89.7 1L2NA/35.0
*C. elegans/ *NP_497960	115–225	FFAS03/COG/SMT3/-11.8	1A5R/27.2
	225–328	FFAS03/COG/SMT3/-15.3	1A5R/38.5 1L2NA/17.6
*M. musculus/ *NP_035030	220–320	RPS-BLAST/SMART/UBQ/3e-05 FFAS03/COG/SMT3/-11	1L2NA/24.3 1A5R/19.9
	320–412	RPS-BLAST/COG/SMT3/1e-04	1L2NA/89.4 1A5R/71.6
*A. nidulans/ *EAA62889	160–300	FFAS03/COG/SMT3/-8.3	1A5R/22.5
	300–404	RPS-BLAST/SMART/UBQ/3e-04 FFAS03/COG/SMT3/-39.100	1A5R/87.4 1L2NA/30.6
*A thaliana/ *NP_564924	130–213	RPS-BLAST/COG/SMT3/4e-06	1L2NA/119.1 1A5R/51.4
*D discoideus/ *JC3V1_0C0008_11033	230–340	RPS-BLAST/COG/SMT3/0.001	1A5R/141.9 1L2NA/57.0
	340–449	FFAS03/COG/SMT3/-33.600	1A5R/51.3 1L2NA/39.4
